# Research progress in PANoptosis mechanisms and their interplay with tuberculosis

**DOI:** 10.3389/fcimb.2026.1786786

**Published:** 2026-03-20

**Authors:** Xue Ma, Yan Liang, Xueqiong Wu, Huiru An

**Affiliations:** 1 Beijing Key Laboratory of New Techniques of Tuberculosis Diagnosis and Treatment, Institute of Tuberculosis Research, Senior Department of Tuberculosis, The Eighth Medical Center of Chinese People's Liberation Army (PLA), General Hospital, Beijing, China; 2 Graduate School, Hebei North University, Zhangjiakou, China

**Keywords:** *Mycobacterium tuberculosis*(M.tb), PANoptosis, PANoptosome, programmed cell death (PCD), tuberculosis(TB)

## Abstract

PANoptosis is a recently identified, inflammatory programmed cell death pathway that amalgamates features of pyroptosis, apoptosis, and necroptosis, executed via multiprotein complexes called PANoptosomes. Increasing evidence highlights PANoptosis has emerged as a potentially pivotal factor in the pathogenesis of several diseases, including viral and bacterial infections and malignancies. Given its extensive pathophysiological relevance, this review systematically explores the conceptual underpinnings, molecular mechanisms, and structural composition of PANoptosome. Special attention is devoted to elucidating the pathophysiological interactions between PANoptosis and tuberculosis (TB), with the aim of developing integrated diagnostic-therapeutic strategies and targeted pharmaceutical innovations for TB. Tuberculosis, a respiratory infectious disease caused by *Mycobacterium tuberculosis(M.tb)*, continues to pose a significant global health challenge.

## 1 Introduction

Tuberculosis (TB), an infectious respiratory disease caused by *Mycobacterium tuberculosis(M.tb)*, continues to pose a substantial global health challenge. As reported in the World Health Organization’s 2024 Global Tuberculosis Report, there were approximately 10.8 million new TB cases and 1.25 million TB-related fatalities worldwide in 2023 (World Health, 2024). The growing prevalence of drug-resistant M.tb strains underscores the urgent need for novel therapeutic strategies (Ernest et al., 2021). A defining histopathological feature of TB is granuloma formation—highly organized aggregates of immune cells, such as macrophages and lymphocytes, that harbor pathogens. Within the complex host-M.tb microenvironment, granulomas perform a dual role: they limit bacterial spread while also creating niches that may facilitate pathogen proliferation through host cell death. As a result, regulated cell death mechanisms are increasingly recognized as critical determinants of TB pathogenesis (Ramakrishnan, 2012).

The innate immune system serves as the primary defense mechanism against infections and cellular disturbances. Upon activation, it triggers inflammatory programmed cell death (PCD) in response to microbial invasion or disruptions in cellular homeostasis. PCD is crucial for host defense and the maintenance of physiological balance (Gullett et al., 2022). Among the well-characterized modalities of PCD, three principal forms are distinguished by their unique mechanisms: Apoptosis is a non-inflammatory, caspase-dependent process. It is regulated through both extrinsic (death receptor) and intrinsic (mitochondrial) pathways. Key molecules include caspases, TNF superfamily members, Bcl-2 proteins, p53, and inhibitor of apoptosis proteins (IAPs) (Levine, 1997; Green, 2019). The extrinsic pathway is initiated by caspase-8 activation, while the intrinsic pathway involves caspase-9. Both converge on the activation of executioner caspases-3/7, leading to DNA fragmentation and cell shrinkage (Kesavardhana et al., 2020b). Pyroptosis is an inflammatory lytic death initiated by inflammasome activation and inflammatory caspases (Malireddi et al., 2020c), Inflammasomes are multiprotein complexes typically composed of sensor proteins and the adaptor ASC, which facilitates the recruitment and activation of caspase-1 (Malik and Kanneganti, 2017). Active caspase-1 cleaves Gasdermin D (GSDMD), releasing its N-terminal domain (GSDMD-NT) that forms pores in the plasma membrane, causing cytokine release and cell lysis (Shi et al., 2015). Necroptosis is a regulated necrotic process primarily mediated by receptor-interacting protein kinase 1 (RIPK1), RIPK3, and their substrate, mixed lineage kinase domain-like protein (MLKL) (Yuan et al., 2019). When caspase-8 activity is inhibited, RIPK1 and RIPK3 form a complex called the necrosome via RHIM domain interactions. This leads to MLKL phosphorylation. Phosphorylated MLKL oligomerizes and translocates to the plasma membrane, disrupting membrane integrity and causing cell lysis (Yuan et al., 2019). Historically considered as distinct pathways, recent evidence has demonstrated significant crosstalk among these programmed cell death (PCD) forms (Tweedell and Kanneganti, 2020). In 2019, Kanneganti’s research group identified PANoptosis, a novel PCD modality that integrates characteristics of pyroptosis (P), apoptosis (A), and necroptosis (N), and is executed via multiprotein complexes known as PANoptosome (Malireddi et al., 2019; Christgen et al., 2022).

This review systematically explores the conceptual underpinnings, molecular mechanisms, and functional implications of PANoptosis, with a particular focus on its association with tuberculosis.

## 2 PANoptosis

### 2.1 Conceptual framework of PANoptosis​​

PANoptosis represents an inflammatory lytic cell death pathway identified in recent years, orchestrated by caspases and members of the RIPK family, and regulated by PANoptosome. It amalgamates essential features of pyroptosis, apoptosis, and/or necroptosis, yet cannot be entirely elucidated by any single pathway alone (Malireddi et al., 2019). Substantial evidence supports the existence of extensive crosstalk among pyroptosis, apoptosis, and necroptosis. In 2016, studies demonstrated that influenza A virus (IAV) infection activates Z-DNA binding protein 1 (ZBP1) through its core nucleoproteins NP and PB1 (Polymerase Basic Protein 1), leading to the assembly of a PANoptosome complex, within which the NLRP3 inflammasome is activated. This process induces simultaneous activation​of apoptosis, necroptosis, and pyroptosis in mouse bone marrow-derived macrophages (BMDMs) via the RIPK1-RIPK3-caspase-8 signaling pathway (Kuriakose et al., 2016), establishing molecular evidence for intrinsic connections between distinct cell death modalities.

#### 2.1.1 Crosstalk between pyroptosis and apoptosis

Gasdermin D (GSDMD), a pore-forming protein of the gasdermin family, mediates pyroptosis execution through its membrane-perforating activity and serves as the primary executioner downstream of caspase-1 and caspase-11. However, in GSDMD-deficient cells, caspase-1 retains cell death-inducing capacity: Tsuchiya et al. demonstrated apoptotic activation via the Bid-caspase-9-caspase-3 axis (Tsuchiya et al., 2019; Kesavardhana et al., 2020b). Furthermore, when GSDMD is absent, caspase-1 can activate apoptotic pathways. It cleaves the protein Bid. Cleaved Bid then causes mitochondrial outer membrane permeabilization (MOMP). This releases pro-apoptotic factors like SMAC. SMAC neutralizes IAPs (Inhibitor of Apoptosis Proteins), allowing caspase-3 to activate. Thus, caspase-1 induces apoptosis when pyroptosis is blocked (Heilig et al., 2020). Also, high levels of caspase-1 can directly cleave and activate other apoptotic proteins, not just its typical pyroptosis targets (Walsh et al., 2011). In other situations, the gasdermin family member GSDME can be involved. Upon TNF or chemotherapeutic stimulation, the family member GSDME undergoes caspase-3-dependent cleavage, liberating its N-terminal domain (GSDME-N) to form membrane pores. This process converts caspase-3-dependent apoptosis to pyroptosis (Wang et al., 2017). Additionally, caspase-11-dependent bile acid-APAF1 apoptosomes activate caspase-3, driving GSDME-mediated pyroptosis (Xu et al., 2021). CARD-domain inflammasome sensors (e.g., NLRP1b and NLRC4) can initiate caspase-1-dependent pyroptosis independently of the ASC adaptor. Van Opdenbosch et al. confirmed that in caspase-1-deficient macrophages and intestinal epithelial organoids (IEOs), NLRP1b/NLRC4 activates compensatory apoptosisthrough caspase-8 (Van Opdenbosch et al., 2017). Beyond regulating extrinsic apoptosis, caspase-8 modulates inflammasome activity by enhancing NLRP3 priming and post-translational modifications (Wu et al., 2014). These mechanisms collectively demonstrate robust molecular crosstalk between pyroptosis and apoptosis.

#### 2.1.2 Crosstalk between necroptosis and apoptosis

RIPK1 serves as a master regulator of TNF-α-induced signaling pathways, coordinating NF-κB activation, apoptosis, and necroptosis (Hsu et al., 1995; Stanger et al., 1995). Caspase-8 acts as a molecular switch between necroptosis and apoptosis: its activation cleaves RIPK1/RIPK3 to inhibit necroptosis while executing apoptosis (Kesavardhana et al., 2020b). Notably, this switch is also regulated by post-translational modifications. For instance, phosphorylation of procaspase-8 can inactivate it within the necrosome, allowing necroptosis to proceed, highlighting the context-dependent regulation of this switch (Yang et al., 2020). Critically, this regulatory phosphorylation of caspase-8, mediated by the kinase RSK, has been directly observed *in vivo*, confirming that the RIPK1-caspase-8 checkpoint is subject to dynamic, context-dependent regulation within physiological settings to bypass its inhibitory function (He et al., 2024). When caspase-8 or FADD is blocked to suppress apoptosis, RIPK1 initiates necroptosis by assembling the RIPK3-MLKL necrosome (Liu et al., 2017). Notably, cells lacking both RIPK1 and caspase-8 exhibit heightened susceptibility to TRIF/IFN-RIPK3-MLKL-mediated necroptosis (Dillon et al., 2014). These findings establish the RIPK1-caspase-8 axis as the core regulatory mechanism underlying necroptosis-apoptosis crosstalk, with more clearly defined molecular pathways than other death modality interactions.

#### 2.1.3 Crosstalk between pyroptosis and necroptosis

The responsive activation of the NLRP3 inflammasome to necroptotic stimuli in cells establishes a molecular linkage between pyroptosis and necroptosis. Studies demonstrate that when caspase activity is inhibited, Toll-like receptor 3 (TLR3) signaling activates the NLRP3 inflammasome through RIPK3-dependent necroptosis (Kang et al., 2015). Notably, during necroptosis, MLKL pore formation induces K^+^ efflux and release of damage-associated molecular patterns (DAMPs), which synergistically induce NLRP3 inflammasome activation [ (Frank and Vince, 2019). The role of RIPK3 in promoting pyroptosis is significant. Evidence shows that in macrophages lacking MLKL and key inflammasome components, necroptotic signaling can trigger a RIPK3-MLKL-NLRP3-caspase-1 axis. This pathway results in the maturation and release of IL-1β (Gutierrez et al., 2017). Furthermore, deficiency in the NLRC4 inflammasome sensor can lead to enhanced necroptosis. For instance, during Pseudomonas aeruginosa infection, NLRC4-deficient mouse bone marrow-derived macrophages (BMDMs) show reduced activation of caspase-1, -3, -7, and -8. This is accompanied by increased activation of necroptotic molecules RIPK1 and MLKL, illustrating a compensatory crosstalk between pyroptosis and necroptosis (Sundaram et al., 2022).

These mechanisms collectively elucidate the interplay between pyroptosis and necroptosis pathways.

### 2.2 Mechanisms of PANoptosis

PANoptosis is regulated by upstream sensors and signaling cascades, which assemble into a multiprotein complex—the PANoptosome (Christgen et al., 2020). However, the precise assembly mechanism based on homotypic domain interactions remains incompletely elucidated. Structurally analogous to inflammasomes, the PANoptosome comprises three core components:

1. Sensor proteins​​ (e.g., ZBP1, NLRP3) that initiate complex formation;
2. Adaptor proteins​​ (e.g., ASC, FADD) that mediate signal transduction;
3. Signal-integrating or licensing proteins​​ (e.g., caspase-1, caspase-8, RIPK3) that regulate downstream terminal effectors (e.g., GSDMD, caspase-3/7, MLKL) (Sundaram and Kanneganti, 2021; Christgen et al., 2022).

These functional classifications are context-dependent. For example, necroptosis requires kinase-active RIPK1 as a catalytic effector, whereas in TAK1 (transforming growth factor-β-activated kinase 1)-deficient cells, kinase-inactive RIPK1 acts as a scaffold to promote NLRP3 inflammasome activation and cell death, indicating its dual role as an adaptor (Malireddi et al., 2020a). This functional plasticity represents a key complexity in PANoptosis research.

Currently identified upstream regulators include ZBP1, AIM2 (absent in melanoma 2), RIPK1, and NLRC5, which trigger PANoptosome assembly upon specific stimuli.

#### 2.2.1 ZBP1 PANoptosome

ZBP1 contains two Z-nucleic acid-binding domains (Zα1 and Zα2) at its N-terminus and two RIP homotypic interaction motifs (RHIM1 and RHIM2) in its central region. The Zα domains detect viral RNA (e.g., IAV), triggering PANoptosome assembly. Deletion of Zα or solely Zα2 completely suppresses PANoptosis (Nagata and Tanaka, 2017; Kesavardhana et al., 2020a). Studies confirm that the ZBP1 PANoptosome comprises ZBP1, RIPK1, RIPK3, caspase-8, caspase-6, caspase-1, ASC, and NLRP3 (Zheng and Kanneganti, 2020a).

ZBP1 recruits RIPK3 via RHIM domain homotypic interactions. RIPK3 then phosphorylates the necroptosis effector MLKL while simultaneously recruiting RIPK1 through RHIM interactions. Subsequently, RIPK1 binds FADD and recruits caspase-8—the core executor of intrinsic apoptosis—activating downstream effectors caspase-3/caspase-7. Concurrently, the adaptor protein ASC engages caspase-8 through heterotypic interactions, recruiting NLRP3 and caspase-1 to induce pyroptosis via GSDMD activation (Malireddi et al., 2019; Zheng and Kanneganti, 2020b; Wang and Kanneganti, 2021; Nguyen and Kanneganti, 2022).

This pathway undergoes precise multi-factor regulation. Interferon regulatory factor 1 (IRF1) drives ZBP1/AIM2/RIPK1/NLRP12-dependent PANoptosis in response to infectious and sterile stimuli (Sharma et al., 2023). Caspase-6 promotes pathway activation by enhancing RIPK3-ZBP1 interactions (Zheng et al., 2020). ADAR1 (adenosine deaminase acting on RNA 1), the only mammalian protein sharing the Zα domain with ZBP1, inhibits PANoptosis through its IFN-inducible p150 isoform, which competes with RIPK3 for ZBP1 binding (Patterson and Samuel, 1995; Karki and Kanneganti, 2023). Beyond protein regulators, ZBP1 isoforms modulate signaling: the murine splice variant ZBP1-S (containing Zα but lacking RHIM) exhibits high IFN sensitivity and inhibits ZBP1 signaling in a Zα-dependent manner (Nagata et al., 2024). Human ZBP1 has ≥7 isoforms, with isoform 5 (Q9H71-5) similarly RHIM-deficient but functionally uncharacterized (Nassour et al., 2023). Whether this human ZBP1-S analog functions similarly to murine ZBP1-S remains unknown. Elucidating the roles of these human short isoforms holds significant implications for developing novel therapeutics targeting ZBP1 to suppress PANoptosis.

#### 2.2.2 AIM2 PANoptosome

The AIM2 PANoptosome contains an N-terminal pyrin domain (PYD) and a C-terminal hematopoietic interferon-inducible nuclear protein (HIN) domain that binds dsDNA. The HIN domain mediates DNA recognition, while the PYD interacts with ASC via PYD-PYD homotypic binding, recruiting caspase-1 through CARD-CARD interactions to assemble the AIM2 inflammasome (Nassour et al., 2023). This complex serves as a core component of the PANoptosome, and its activation is essential for pyroptosis and PANoptosis (Sundaram and Kanneganti, 2021). Among the five known inflammasome sensors (NLRP1, NLRP3, AIM2, NLRC4, and pyrin), the latter four participate in PANoptosis.

The AIM2 PANoptosome comprises AIM2, ZBP1, pyrin, ASC, caspase-1, caspase-8, caspase-6, RIPK3, RIPK1, and FADD. It plays critical roles in human development, viral infections, inflammation, and cancer by recognizing double-stranded DNA. AIM2 deficiency reduces pyrin and ZBP1 expression, indicating that AIM2 acts as an upstream regulator controlling PANoptosome assembly and activation (Lee et al., 2021).

Pathogens such as herpes simplex virus 1 (HSV1) and *Francisella novicida*not only activate AIM2 but also interact with the Zα domain of ZBP1 while inhibiting Rho-GTP activity, thereby driving the formation of the AIM2-pyrin-ZBP1 PANoptosome (Lee et al., 2021). Beyond IRF1, eukaryotic translation initiation factor 2 alpha kinase 2 (EIF2AK2) regulates AIM2 inflammasome activation (Varghese et al., 2015). Inhibition of EIF2AK2 reduces AIM2 activation (Xie et al., 2025), while Wei et al. demonstrated that EIF2AK2 induces AIM2-dependent PANoptosis in septic kidney injury, exacerbating renal damage (Wei et al., 2024).

#### 2.2.3 RIPK1 PANoptosome

The RIPK1 PANoptosome contains three structural domains: an N-terminal kinase domain, a RIP homotypic interaction motif (RHIM), and a C-terminal death domain (DD). Its serine/threonine kinase activity plays a central role in necroptosis and RIPoptosome-mediated caspase-dependent apoptosis (Sun et al., 2002). This PANoptosome was identified during Yersinia infection and comprises RIPK1, NLRP3,ASC, caspase-1, caspase-8, and FADD, with assembly regulated by TAK1 (transforming growth factor β-activated kinase 1) (Malireddi et al., 2020a). In TAK1-deficient cells, kinase-inactive RIPK1 can act as a scaffold to promote NLRP3 inflammasome activation and cell death, indicating its dual role as a sensor and adaptor (Malireddi et al., 2020a). Under physiological conditions, TAK1 inhibits RIPK1 phosphorylation to block PANoptosis activation (Geng et al., 2017). Conversely, the Yersinia toxin YopJ inactivates TAK1 (Mukherjee et al., 2006), thereby promoting TAK1-deficient cells to form RIPK1-caspase-8 complexes that trigger RIPK1-dependent PANoptosis (Malireddi et al., 2019). Furthermore, in the absence of TAK1, a RIPK1-independent PANoptosis pathway can be activated, which is mediated by RIPK3 and MLKL (Malireddi et al., 2020a).

While compositionally similar to the ZBP1 PANoptosome, this complex uniquely employs RIPK1 as the sensor instead of ZBP1 (Lee et al., 2021) Notably, RIPK1 deficiency fails to fully prevent cell death but instead promotes necroptosis while attenuating *Yersinia*-induced pyroptosis and apoptosis (Malireddi et al., 2020b).

Given the centrality of TAK1 inhibition (TAK1i)-induced cell death in PANoptosis, elucidating its regulatory mechanisms is critical. Two classes of key regulators have been identified: RNA-binding proteins polypyrimidine tract-binding (PTB) protein 1 (PTBP1) and RAVER1 maintain functional RIPK1 expression by suppressing aberrant *Ripk1*pre-mRNA splicing, with knockdown experiments confirming their depletion significantly reduces inflammasome activation and core PANoptosis pathway activity (Malireddi et al., 2023). While within the phosphatase 6(PP6) holoenzyme complex, loss of the catalytic subunit PPP6C (protein phosphatase 6 catalytic subunit) strongly inhibits TAK1i-induced PANoptosis, whereas regulatory subunits (PPP6R1/R2/R3) require triple knockout to block cell death, demonstrating functional redundancy among these subunits. The dominant effect of PPP6C loss highlights the essential role of phosphatase activity, while the redundancy among regulatory subunits suggests any one of them can recruit the catalytic subunit to its substrates (Bynigeri et al., 2024).

#### 2.2.4 NLRC5-NLRP12 PANoptosome

NLRC5 contains an N-terminal CARD domain, a central nucleotide-binding domain (NBD), and a C-terminal leucine-rich repeat (LRR) domain. Expressed in both innate and adaptive immunity, it activates the PANoptosome in response to heme and pathogen-associated molecular patterns (PAMPs), inducing inflammatory cell death (Kobayashi and van den Elsen, 2012). NLRP12, characterized by LRR, NBD, and N-terminal protein interaction domains, was first identified in human leukemia cell lines (de Lima et al., 2023). It acts as a sensor during Yersinia pestisor Plasmodium chabaudi infection, driving pyroptosis via the ASC-caspase-1 inflammasome. Notably, NLRP12 suppresses human NLRP3 inflammasome assembly—a human-specific phenomenon not observed in mice (Coombs et al., 2024).

Upon stimulation by heme combined with PAMPs or TNF, TLR2/4-MyD88 signaling induces IRF1 expression and ROS generation. This upregulates NLRP12 expression and initiates PANoptosome assembly (containing NLRP12, caspase-8, ASC, and RIPK3), ultimately executing PANoptosis. Further studies reveal that under identical stimuli, NLRC5 co-localizes with ASC, caspase-8, and RIPK3 to form a multiprotein complex strictly dependent on NLRC5, NLRP12, and ASC (Sundaram et al., 2023, 2024). Crucially, while NLRC5 and NLRP12 cooperatively drive PANoptosis, their activation pathways differ fundamentally: NLRC5 upregulation occurs via the TLR2/4-NAD^+^ axis (ROS-independent), whereas NLRP12 activation requires ROS generation (Sundaram et al., 2024). The assembly mechanism and direct intermolecular interactions within the NLRC5-NLRP12 PANoptosome remain uncharacterized, necessitating further investigation into its molecular architecture and regulatory networks.

**Figure 1:**
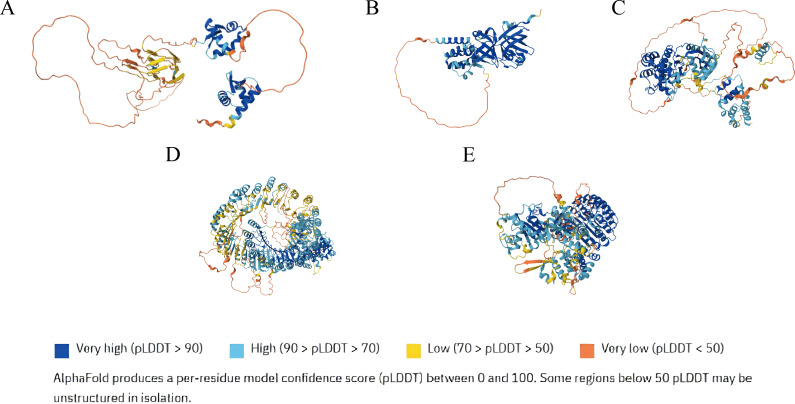
The structure of panoptosis upstream regulatory factors **(A)** ZBP1 Protein Structure **(B)** AIM2 Protein Structure **(C)** RIPK1 Protein Structure **(D)** NLRC5 Protein Structure **(E)** NLRP12 Protein Structure.

Based on these findings, **Figure 1** summarizes predicted structural features of PANoptosis upstream regulators (Varadi et al., 2024), while **Table 1** details components and regulators of individual PANoptosome.

**Table 1:** PANoptosome and their regulatory factors.

PANoptosome	Component	Regulatory factors	References
ZBP1	ZBP1, RIPK1, RIPK3, CASP8, CASP6, CASP1, ASC and NLRP3	IRF1,ADAR1,CASP6	(Zheng and Kanneganti, 2020a; Zheng et al., 2020; Karki and Kanneganti, 2023; Sharma et al., 2023)
AIM2	AIM2, ZBP1, pyrin, ASC, CASP1, CASP8, RIPK3, and FADD	IRF1, EIF2AK2	(Lee et al., 2021; Sharma et al., 2023)
RIPK1	RIPK1,RIPK3 ASC, CASP1, CASP8, and FADD	IRF1,TAK1、PTB1、RAVER1、PP6	(Geng et al., 2017; Malireddi et al., 2020a, 2023; Sharma et al., 2023; Bynigeri et al., 2024)
NLRC5-NLRP12	NLRC5,NLRP12, CASP8, ASC, NLRP3andRIPK3	IRF1,TLR2, TLR4	(Nguyen and Kanneganti, 2022; Sharma et al., 2023)

## 3 Immunological mechanisms of macrophages in tuberculosis infection

Macrophages serve as primary host cells for M.tb and as key effectors coordinating innate and adaptive immunity (Marakalala et al., 2018). Upon recognizing M.tb via surface pattern recognition receptors (PRRs, e.g., TLR4), they activate NOX2-mediated phagocytic killing to initiate innate defense (Lv et al., 2017). Concurrently, secretion of cytokines (IL-12, TNF-α) drives Th1-polarized immunity, bridging adaptive responses (Zhang et al., 2024). However, M.tb hijacks intracellular iron and fatty acids to sustain replication, forcing infected cells to upregulate anti-inflammatory factors (e.g., IL-10) that counteract bacterial proliferation, resulting in intense nutritional competition (Lee et al., 2020).

To evade immune clearance, M.tb employs dual escape strategies: suppressing host apoptosis and autophagy to block antimicrobial programs (Zhai et al., 2019), while secreting virulence factor ESAT-6 to induce oxidative stress, causing DNA damage and impairing macrophage bactericidal function (Mohanty et al., 2016). This dynamic host-pathogen interplay drives macrophage metabolic reprogramming—infected cells undergo M1 polarization, relying on glycolysis and the pentose phosphate pathway (PPP) for energy. Critically, this metabolic shift modulates ROS production and cytokine secretion, profoundly impacting antibacterial efficacy and disease outcomes (Kumar et al., 2019).

Notably, macrophage death modalities (apoptosis, pyroptosis, necroptosis) directly determine TB immunopathology. While M.tb traditionally inhibits apoptosis/autophagy to maintain intracellular survival, emerging evidence reveals that PANoptosis—a coordinated death program integrating pyroptosis, apoptosis, and necroptosis—may be activated in M.tb-infected macrophages, breaching pathogen immune evasion. This novel pathway, executed through PANoptosome​that integrate multiple signals, offers promising therapeutic targets for TB immunotherapy.

## 4 Relationship between PANoptosis and tuberculosis

### 4.1 Relationship between apoptosis and tuberculosis

In TB, apoptosis serves as a critical cell death modality with complex immunoregulatory roles. M.tb employs multifaceted mechanisms to modulate host apoptosis, sustaining intracellular survival and promoting dissemination. Studies demonstrate that M.tb bidirectionally regulates host apoptosis to influence disease progression (Behar et al., 2011; Lam et al., 2017).

Apoptosis functions as a key innate immune defense mechanism, effectively restricting M.tb proliferation and limiting pathogen spread. Evidence indicates that apoptotic macrophages in M.tb infection exhibit significantly reduced bacterial viability (Behar et al., 2011). This process further enhances adaptive immunity by activating CD8^+^ T-cell responses (Jurcic Smith and Lee, 2016).

However, M.tb has evolved strategies to inhibit apoptosis, prolonging intracellular survival. Secreted proteins Rv3654c and Rv3655c target host factors PSF (splicing factor) and ALOX17 (lipoxygenase), disrupting extrinsic apoptotic pathways (Danelishvili et al., 2010), The virulence factor Rv3033 suppresses intrinsic apoptosis to promote bacterial persistence (Zhang et al., 2018). Furthermore, M. tb employs additional virulence factors to inhibit host cell death. Notably, the NADH:ubiquinone oxidoreductase subunit G (NuoG)was the first bacterial factor identified to directly inhibit apoptosis of infected host cells (Velmurugan et al., 2007). Significantly, the serine/threonine protein kinase E(PknE)has been shown to play a key role in the bacterial response to nitric oxide stress and in inhibiting apoptosis in a human macrophage model of infection, highlighting its importance in bacterial survival (Jayakumar et al., 2008). M.tb also upregulates IRAK-M expression in macrophages, impeding M1 polarization and weakening immune clearance (Shen et al., 2017). Additionally, M.tb modulates miRNA expression (e.g., miR-1178 upregulation) to inhibit TLR4 signaling and reduce pro-inflammatory cytokine production (Shi et al., 2018).

Notably, certain M.tb virulence factors like ESAT-6 can induce apoptosis through multiple pathways. ESAT-6 activates the intrinsic apoptotic pathway via caspase-9/caspase-3 activation in macrophages (Lin et al., 2019). It also promotes apoptosis through the miRNA-155-SOCS1 regulatory axis (Yang et al., 2015). These mechanisms reflect M.tb’s sophisticated manipulation of host apoptosis to facilitate dissemination and chronic infection.

### 4.2 Relationship between pyroptosis and tuberculosis

Pyroptosis is a lytic form of programmed cell death mediated by the cleavage of gasdermin family proteins (e.g., GSDMD), which can be triggered by multiple inflammatory caspases (Broz et al., 2020). This process relies on intracellular iron depletion to induce macrophage necrosis, constituting a critical host defense mechanism (Dawi et al., 2025). During M.tb infection, pathogen/damage-associated molecular patterns (PAMPs/DAMPs) activate the NLRP3 inflammasome, triggering caspase cascades. Activated caspases not only promote maturation of pro-inflammatory cytokines IL-1β/IL-18 but also cleave GSDMD to execute pyroptosis—enhancing anti-infective immunity while potentially causing tissue damage and pathogen dissemination (Theobald et al., 2024). Notably, M.tb counteracts by secreting phosphatase PtpB, which dephosphorylates host membrane phospholipids (PI4P/PIP2), disrupting membrane localization of cleaved GSDMD and thereby suppressing cytokine release and pyroptosis (Chai et al., 2022).

To overcome this immune evasion, studies reveal that baicalin inhibits PERK/eIF2α signaling to reduce TXNIP (Thioredoxin Interacting Protein) expression and NLRP3 inflammasome activation, consequently suppressing pyroptosis in M.tb-infected macrophages (Fu et al., 2021). Additionally, TLR4-mediated endoplasmic reticulum stress (ERS) represents another key regulatory pathway: BCG infection activates TLR4 signaling to induce ERS and NLRP3 activation. Paradoxically, inhibiting this pathway attenuates pyroptosis but exacerbates lung injury and promotes pathogen survival (Nie et al., 2025).

### 4.3 Relationship between necroptosis and tuberculosis

Necroptosis, a programmed cell death modality, is closely associated with M.tb)infection. Studies demonstrate that macrophages undergo lytic cell death during late infection stages or under high multiplicity of infection (MOI) conditions (Lee et al., 2006; Park et al., 2006). Consistent with this, zebrafish infection models confirm that M.tb-infected macrophages experience TNF-induced necroptotic lysis (Roca and Ramakrishnan, 2013). A hypothesis suggests this death modality may benefit mycobacterial survival by releasing bacteria into growth-favorable extracellular environments (Tobin et al., 2012). Stutz et al. recently revealed through murine models that M.tb-infected macrophages remodel intracellular signaling by upregulating MLKL, TNFR1, and ZBP1 while downregulating cIAP1, creating a pro-necroptotic microenvironment. Paradoxically, genetic knockout (*Mlkl*^-^/^-^) or RIPK1 inhibition (Nec-1s) failed to affect survival of infected human/murine macrophages. In humanized mouse models with MLKL deficiency or Nec-1s treatment, lung histopathology and bacterial burden showed no significant differences versus controls (Stutz et al., 2018).

These findings indicate that while M.tb infection initiates macrophage necroptotic pathways, this signaling cascade is physiologically abrogated, diminishing its pathophysiological contribution to TB-related lung injury.

### 4.4 Summary and perspectives on PANoptosis in tuberculosis

**Figure 2:**
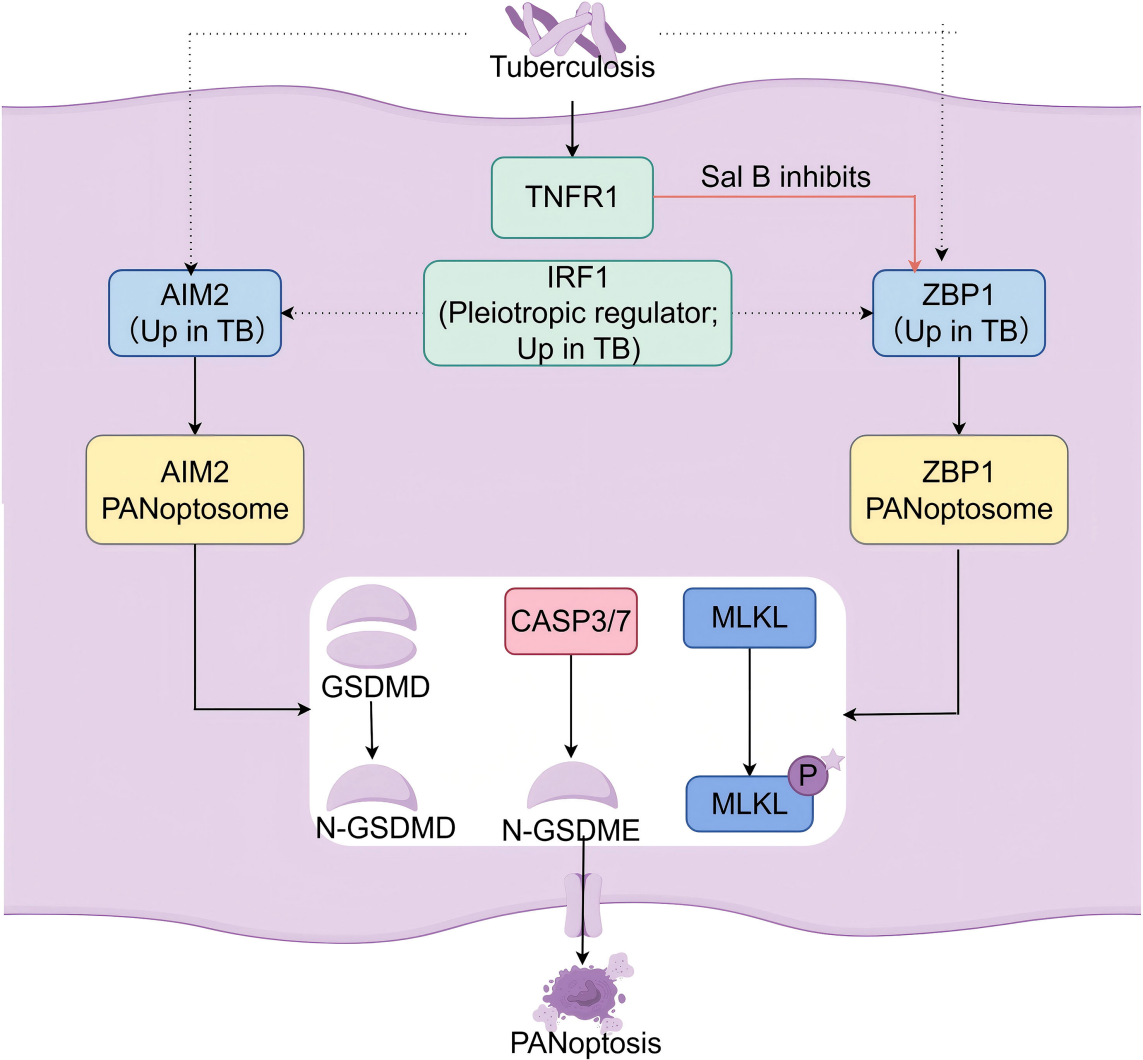
A proposed model for PANoptosis activation in Mycobacterium tuberculosis-infected macrophages.This schematic illustrates a hypothesized signaling network through which M. tuberculosis (M.tb) infection may trigger PANoptosis, an integrated inflammatory cell death pathway. The model integrates transcriptional upregulation data from TB infection with known molecular interactions from related pathophysiological contexts. Key components and annotations: Solid arrows denote molecular interactions with direct experimental support in other models. Dashed arrows indicate connections inferred specifically for the TB context, based on gene expression data and pathway homology. The pleiotropic regulator IRF1 and the sensor proteins ZBP1 and AIM2 are highlighted as upregulated in TB. The TNFR1-ZBP1 axis is included, with inhibition by Salvianolic Acid B (Sal B) indicated, representing a potential therapeutic target. The necroptosis executor MLKL is marked with an asterisk (*), signifying that its activation (phosphorylation, MLKL-P) may be “primed but contextually restrained” during M.tb infection, contributing to signal integration rather than executing full necroptosis. The model proposes that M.tb infection engages upstream sensors (e.g., ZBP1, AIM2) and regulators (e.g., IRF1, TNFR1), leading to the assembly of distinct PANoptosome complexes. These complexes coordinate the activation of terminal effectors for pyroptosis (GSDMD cleavage), apoptosis (Caspase-3/7 activation), and necroptosis (MLKL phosphorylation), culminating in PANoptosis.

Integrated analysis of peripheral blood transcriptomes from tuberculosis patients using GEO2R reveals a significant upregulation of AIM2 and ZBP1 across several datasets (**Table 2**). In line with this, previous work has reported elevated expression of the key PANoptosis regulator IRF1 in datasets GSE19491 and GSE50834, a finding validated by ELISA, further supporting the involvement of PANoptosis-related factors in TB (Zhou et al., 2019). Given that IRF1 deficiency increases host susceptibility to pathogens (Langlais et al., 2016), these findings lead us to propose a testable hypothesis and molecular model:*M. tb* infection induces macrophage PANoptosis to restrict bacterial dissemination.In this model, upstream signaling events (e.g., via TNFR1) and pathogen sensing by ZBP1 and AIM2 converge to trigger the assembly of distinct PANoptosomes—the ZBP1 complex (containing ZBP1, NLRP3, ASC, caspases-1/6/8, and RIPK1) and the AIM2 complex (incorporating AIM2, pyrin, ZBP1, ASC, caspases-1/6/8, FADD, and RIPK1/3). These complexes execute PANoptosis through three convergent pathways: caspase-3/7 activation, GSDMD/GSDME cleavage, and MLKL phosphorylation, culminating in membrane pore formation and cell lysis (**Figure 2**).The pleiotropic regulator IRF1, which is upregulated in TB, may create a permissive state that facilitates PANoptosome assembly,while apoptosis-necroptosis switching undergoes dynamic regulation by infection progression and host-pathogen interactions.

**Table 2:** Expression of ZBP1 and AIM2 in transcriptome data.

Data set	Sample type	ZBP1		AIM2	
		UP/DOWN	LogFC	UP/DOWN	LogFC
GSE83456	Blood	UP	1.237454074	UP	1.11186441682722
GSE19444	Blood	UP	0.9506513	UP	1.9522318
GSE19439	Blood	UP	0.79438976	UP	0.79438976
GSE42830	Blood	UP	1.329864624	UP	0.562189513
GSE81746	Blood	UP	0.720873533333336	UP	2.4335261

Furthermore, preliminary experimental support for this model is emerging. A recent study demonstrated that M.tb infection in macrophages enhances the protein level of ZBP1 and promotes the physical interaction among core PANoptosome components, including ASC, ZBP1, RIPK1, RIPK3, and caspase-8. Critically, the natural compound Salvianolic Acid B (Sal B) was shown to inhibit these interactions and subsequent PANoptosis, providing direct functional evidence for the existence and activity of a ZBP1-centered PANoptosome in this context (Shen et al., 2025).

Importantly, the observation of ‘primed but abortive’ necroptotic signaling in TB models is not inconsistent with this integrated paradigm (Stutz et al., 2018).​ Rather, it may reflect precise contextual regulation within the PANoptosis framework. Here, necroptotic components like RIPK1 and RIPK3 can be recruited to the PANoptosome, where they serve essential scaffolding or regulatory roles that influence the activation of pyroptotic and apoptotic effectors, without necessarily culminating in MLKL-dependent lytic cell death under all conditions. This perspective aligns with the view that PANoptosis is not merely the sum of three deaths, but a dynamically tunable response.

While apoptosis, pyroptosis, and necroptosis each contribute distinctly to TB immunopathology, their functions during infection are not isolated. The complex granuloma microenvironment likely provides concurrent signals that engage multiple cell death sensors simultaneously (Ramakrishnan, 2012). PANoptosis embodies the integrated molecular response to such signals, wherein sensing converges onto specific PANoptosomes (Christgen et al., 2020; Lee et al., 2021), which then orchestrate the co-activation of apoptotic, pyroptotic, and necroptotic effector modules within a unified program (Malireddi et al., 2019; Wang and Kanneganti, 2021). Consequently, the documented “bidirectional regulation” of apoptosis by M. tb (Behar et al., 2011; Lam et al., 2017), its suppression of pyroptosis via PtpB (Chai et al., 2022), and the abortive necroptotic signaling (Stutz et al., 2018) may represent pathogen engagements with different facets of this broader PANoptosis process. Therefore, we propose that PANoptosis serves as a compelling central regulatory hub—a cohesive framework that best explains the crosstalk among individual death modalities during TB infection. Investigating TB through this integrative lens, rather than through isolated pathways, provides a more holistic understanding of how host cell fate decisions determine the balance between bacterial control and pathological tissue damage.

### 4.5 Therapeutic strategies targeting PANoptosis in tuberculosis

As an integrated programmed cell death pathway, core components of PANoptosis—such as sensors ZBP1/AIM2, regulators IRF1/TAK1, and signaling amplifiers like Caspase-6—have emerged as promising targets for developing host-directed therapies (HDT) against tuberculosis. ​Direct evidence comes from the natural compound Salvianolic Acid B (Sal B), which effectively inhibits ZBP1-dependent PANoptosis in M.tb-infected macrophages. Mechanistically, Sal B acts by directly antagonizing tumor necrosis factor receptor 1 (TNFR1), leading to the downregulation of the downstream sensor ZBP1 and consequent disruption of PANoptosome assembly (Shen et al., 2025). This action helps maintain host cell viability while restricting bacterial replication, providing a compelling proof of concept for repurposing natural compounds as precise PANoptosis modulators.

Beyond directly targeting death receptors, intervention at upstream regulatory nodes of PANoptosis also shows potential. The pleiotropic transcription factor IRF1, which can regulate the expression of multiple PANoptosome components, increases host susceptibility to infection when deficient (Sharma et al., 2023). This suggests that modulating IRF1 activity could influence inflammatory outcomes, but its broad role in antibacterial immunity makes its therapeutic window narrow and requires careful evaluation. In contrast, inhibition of the kinase TAK1 has been shown to trigger RIPK1-dependent PANoptosis (Malireddi et al., 2020a). Thus, in specific infection contexts, using TAK1 agonists or stabilizers could exert protective effects by maintaining the inhibitory phosphorylation of RIPK1 and preventing its aberrant activation.

At the level of signaling execution, Caspase-6 has been identified as a key amplifier of ZBP1–NLRP3 inflammasome activation and PANoptosis, promoting the interaction between RIPK3 and ZBP1 (Zheng et al., 2020). This makes Caspase-6 a highly attractive target for pharmacological intervention. Additionally, adenosine deaminase ADAR1 acts as an endogenous brake on PANoptosis by competitively binding RNA with ZBP1 (Karki and Kanneganti, 2023). Enhancing ADAR1’s editing activity or stability may represent an alternative therapeutic strategy aimed at boosting the host’s own regulatory mechanisms.

In summary, current explorations of therapeutic strategies targeting PANoptosis in tuberculosis focus on three main directions: (1) targeting death receptors (e.g., TNFR1) or upstream sensors; (2) modulating key signaling nodes (e.g., the pleiotropic IRF1 or the regulatory kinase TAK1); and (3) inhibiting effector amplifiers (e.g., Caspase-6). Future challenges lie in accurately evaluating the efficacy and safety of these interventions within the complex infection microenvironment and in developing delivery systems that can precisely target infection sites while avoiding systemic immunosuppression.

## 5 Discussion

This review explores PANoptosis—a recently defined programmed cell death pathway that converges pyroptosis, apoptosis, and necroptosis—and evaluates its emerging significance as a unifying framework to understand TB immunopathology. We propose that viewing the complex cell death events during M.tb infection through the lens of PANoptosis, rather than as isolated pathways, provides a more dynamic and holistic understanding of host-pathogen interactions. We should note, however, that while this framework is powerfully explanatory, direct experimental evidence for full PANoptosome assembly and function in TB specifically remains an active area of investigation, as outlined in our proposed model (**Figure 2**).

The complex granuloma microenvironment in TB (Ramakrishnan, 2012)—creates conditions that can engage multiple pattern-recognition receptors like ZBP1 and AIM2. We suggest that the apoptosis, pyroptosis, and necroptosis observed during infection are interconnected aspects of a unified host response program. For example, M.tb employs PtpB to inhibit GSDMD-mediated pyroptosis (Chai et al., 2022), while its virulence factor ESAT-6 can trigger apoptosis (Yang et al., 2015; Lin et al., 2019). These seemingly opposing strategies may reflect the pathogen’s adaptation to counteract an integrated host death response—namely, PANoptosis. Likewise, the incomplete or “abortive” necroptotic signaling seen in infection models (Tobin et al., 2012) implies that, *in vivo*, this pathway is likely modulated within the broader PANoptosis network. Together, this integrated perspective moves us beyond a view of separate pathways to a more coherent understanding of host–pathogen interactions in TB.

Key components of the PANoptosis pathway represent promising targets for new host-directed therapies (HDTs).Strategies can be envisioned at several levels: directly targeting upstream sensors or receptors is one viable approach. The natural compound Salvianolic Acid B, for instance, inhibits ZBP1-dependent PANoptosis in M.tb-infected macrophages by engaging TNFR1, thereby preserving host cell viability while limiting bacterial growth (Shen et al., 2025). This highlights the potential to repurpose natural compounds as precise PANoptosis modulators. Modulating central signaling nodes offers another strategic avenue. IRF1 acts as a positive regulator of several PANoptosomes; its deficiency increases infection susceptibility (Langlais et al., 2016; Sharma et al., 2023). Conversely, inhibiting TAK1 kinase activity triggers RIPK1-dependent PANoptosis (Malireddi et al., 2020a). These findings suggest that fine-tuning IRF1 activity or using TAK1-stabilizing agents to maintain RIPK1 in an inactive state could help balance inflammatory cell death with protective immunity, though therapeutic windows require need careful definition. At the level of key amplifiers, Caspase-6, which amplifies ZBP1–NLRP3 inflammasome activation and PANoptosis (Zheng et al., 2020), is a compelling drug target. Simultaneously, boosting endogenous regulators like ADAR1—which acts as a “molecular brake” by competing with ZBP1 for RNA binding (Karki and Kanneganti, 2023)—could strengthen the host’s innate capacity to restrain excessive death signaling.

Translating PANoptosis-directed strategies into clinical tools faces several hurdles. First, the proposed model itself needs rigorous validation. We need better models—such as conditional knockout animals and cell-type-specific interventions—to define the precise roles of different PANoptosome complexes (e.g., ZBP1, AIM2) across infection stages and tissues, clarifying whether they aid bacterial clearance or drive tissue injury. Second, delivering therapeutics precisely to infection sites (e.g., lung granulomas) remains a major challenge. Advances in targeted delivery systems will be essential to maximize local drug activity while minimizing systemic side effects like immunosuppression. Lastly, the framework’s scope needs to be tested. It is still unclear whether newer forms of regulated cell death, such as ferroptosis, are functionally integrated into the PANoptosis network. Techniques like single-cell and spatial transcriptomics could help map the landscape of cell death heterogeneity within TB lesions, potentially revealing new regulatory nodes and therapeutic opportunities.

## 6 Conclusion

The PANoptosis paradigm offers a powerful and unified framework that significantly advances our understanding of cell death in TB. It effectively explains observed pathway crosstalk, reconciles seemingly disparate pathogen strategies, and systematically identifies novel therapeutic targets. By shifting the perspective from isolated pathways to an integrated network, this framework not only clarifies existing data but also provides a clear roadmap for future research aimed at developing innovative host-directed strategies to combat tuberculosis.

## References

[B1] Behar S. M. Martin C. J. Booty M. G. Nishimura T. Zhao X. Gan H. X. . (2011). Apoptosis is an innate defense function of macrophages against Mycobacterium tuberculosis. Mucosal Immunol. 4, 279–287. doi: 10.1038/mi.2011.3, PMID: 21307848 PMC3155700

[B2] Broz P. Pelegrín P. Shao F. (2020). The gasdermins, a protein family executing cell death and inflammation. Nat. Rev. Immunol. 20, 143–157. doi: 10.1038/s41577-019-0228-2, PMID: 31690840

[B3] Bynigeri R. R. Malireddi R. K. S. Mall R. Connelly J. P. Pruett-Miller S. M. Kanneganti T. D. (2024). The protein phosphatase PP6 promotes RIPK1-dependent PANoptosis. BMC Biol. 22, 122. doi: 10.1186/s12915-024-01901-5, PMID: 38807188 PMC11134900

[B4] Chai Q. Yu S. Zhong Y. Lu Z. Qiu C. Yu Y. . (2022). A bacterial phospholipid phosphatase inhibits host pyroptosis by hijacking ubiquitin. Science 378, eabq0132. doi: 10.1126/science.abq0132, PMID: 36227980

[B5] Christgen S. Tweedell R. E. Kanneganti T. D. (2022). Programming inflammatory cell death for therapy. Pharmacol. Ther. 232, 108010. doi: 10.1016/j.pharmthera.2021.108010, PMID: 34619283 PMC8930427

[B6] Christgen S. Zheng M. Kesavardhana S. Karki R. Malireddi R. K. S. Banoth B. . (2020). Identification of the PANoptosome: A molecular platform triggering pyroptosis, apoptosis, and necroptosis (PANoptosis). Front. Cell Infect. Microbiol. 10. doi: 10.3389/fcimb.2020.00237, PMID: 32547960 PMC7274033

[B7] Coombs J. R. Zamoshnikova A. Holley C. L. Maddugoda M. P. Teo D. E. T. Chauvin C. . (2024). NLRP12 interacts with NLRP3 to block the activation of the human NLRP3 inflammasome. Sci. Signal 17, eabg8145. doi: 10.1126/scisignal.abg8145, PMID: 38261657

[B8] Danelishvili L. Yamazaki Y. Selker J. Bermudez L. E. (2010). Secreted Mycobacterium tuberculosis Rv3654c and Rv3655c proteins participate in the suppression of macrophage apoptosis. PloS One 5, e10474. doi: 10.1371/journal.pone.0010474, PMID: 20454556 PMC2864267

[B9] Dawi J. Affa S. Kafaja K. Misakyan Y. Kades S. Dayal S. . (2025). The role of ferroptosis and cuproptosis in tuberculosis pathogenesis: implications for therapeutic strategies. Curr. Issues Mol. Biol. 47, 99. doi: 10.3390/cimb47020099, PMID: 39996820 PMC11853893

[B10] de Lima J. D. de Paula A. G. P. Yuasa B. S. de Souza Smanioto C. C. da Cruz Silva M. C. Dos Santos P. I. . (2023). Genetic and epigenetic regulation of the innate immune response to gout. Immunol. Invest. 52, 364–397. doi: 10.1080/08820139.2023.2168554, PMID: 36745138

[B11] Dillon C. P. Weinlich R. Rodriguez D. A. Cripps J. G. Quarato G. Gurung P. . (2014). RIPK1 blocks early postnatal lethality mediated by caspase-8 and RIPK3. Cell 157, 1189–1202. doi: 10.1016/j.cell.2014.04.018, PMID: 24813850 PMC4068710

[B12] Ernest J. P. Strydom N. Wang Q. Zhang N. Nuermberger E. Dartois V. . (2021). Development of new tuberculosis drugs: translation to regimen composition for drug-sensitive and multidrug-resistant tuberculosis. Annu. Rev. Pharmacol. Toxicol. 61, 495–516. doi: 10.1146/annurev-pharmtox-030920-011143, PMID: 32806997 PMC7790895

[B13] Frank D. Vince J. E. (2019). Pyroptosis versus necroptosis: similarities, differences, and crosstalk. Cell Death Differ 26, 99–114. doi: 10.1038/s41418-018-0212-6, PMID: 30341423 PMC6294779

[B14] Fu Y. Shen J. Li Y. Liu F. Ning B. Zheng Y. . (2021). Inhibition of the PERK/TXNIP/NLRP3 axis by baicalin reduces NLRP3 inflammasome-mediated pyroptosis in macrophages infected with mycobacterium tuberculosis. Mediators Inflammation 2021, 1805147. doi: 10.1155/2021/1805147, PMID: 34790063 PMC8592748

[B15] Geng J. Ito Y. Shi L. Amin P. Chu J. Ouchida A. T. . (2017). Regulation of RIPK1 activation by TAK1-mediated phosphorylation dictates apoptosis and necroptosis. Nat. Commun. 8, 359. doi: 10.1038/s41467-017-00406-w, PMID: 28842570 PMC5572456

[B16] Green D. R. (2019). The coming decade of cell death research: five riddles. Cell 177, 1094–1107. doi: 10.1016/j.cell.2019.04.024, PMID: 31100266 PMC6534278

[B17] Gullett J. M. Tweedell R. E. Kanneganti T. D. (2022). It’s all in the PAN: crosstalk, plasticity, redundancies, switches, and interconnectedness encompassed by PANoptosis underlying the totality of cell death-associated biological effects. Cells 11, 1495. doi: 10.3390/cells11091495, PMID: 35563804 PMC9105755

[B18] Gutierrez K. D. Davis M. A. Daniels B. P. Olsen T. M. Ralli-Jain P. Tait S. W. . (2017). MLKL activation triggers NLRP3-mediated processing and release of IL-1β Independently of gasdermin-D. J. Immunol. 198, 2156–2164. doi: 10.4049/jimmunol.1601757, PMID: 28130493 PMC5321867

[B19] He P. Ai T. Qiao M. Yang Z. H. Han J. (2024). Phosphorylation of caspase-8 by RSKs via organ-constrained effects controls the sensitivity to TNF-induced death. Cell Death Discov. 10, 255. doi: 10.1038/s41420-024-02024-0, PMID: 38789425 PMC11126741

[B20] Heilig R. Dilucca M. Boucher D. Chen K. W. Hancz D. Demarco B. . (2020). Caspase-1 cleaves Bid to release mitochondrial SMAC and drive secondary necrosis in the absence of GSDMD. Life Sci. Alliance 3, e202000735. doi: 10.26508/lsa.202000735, PMID: 32345661 PMC7190276

[B21] Hsu H. Xiong J. Goeddel D. V. (1995). The TNF receptor 1-associated protein TRADD signals cell death and NF-kappa B activation. Cell 81, 495–504. doi: 10.1016/0092-8674(95)90070-5, PMID: 7758105

[B22] Jayakumar D. Jacobs W. R. Jr. Narayanan S. (2008). Protein kinase E of Mycobacterium tuberculosis has a role in the nitric oxide stress response and apoptosis in a human macrophage model of infection. Cell Microbiol. 10, 365–374. doi: 10.1111/j.1462-5822.2007.01049.x, PMID: 17892498

[B23] Jurcic Smith K. L. Lee S. (2016). Inhibition of apoptosis by Rv2456c through Nuclear factor-κB extends the survival of Mycobacterium tuberculosis. Int. J. Mycobacteriol 5, 426–436. doi: 10.1016/j.ijmyco.2016.06.018, PMID: 27931684 PMC5975360

[B24] Kang S. Fernandes-Alnemri T. Rogers C. Mayes L. Wang Y. Dillon C. . (2015). Caspase-8 scaffolding function and MLKL regulate NLRP3 inflammasome activation downstream of TLR3. Nat. Commun. 6, 7515. doi: 10.1038/ncomms8515, PMID: 26104484 PMC4480782

[B25] Karki R. Kanneganti T. D. (2023). ADAR1 and ZBP1 in innate immunity, cell death, and disease. Trends Immunol. 44, 201–216. doi: 10.1016/j.it.2023.01.001, PMID: 36710220 PMC9974732

[B26] Kesavardhana S. Malireddi R. K. S. Burton A. R. Porter S. N. Vogel P. Pruett-Miller S. M. . (2020a). The Zα2 domain of ZBP1 is a molecular switch regulating influenza-induced PANoptosis and perinatal lethality during development. J. Biol. Chem. 295, 8325–8330. doi: 10.1074/jbc.RA120.013752, PMID: 32350114 PMC7294087

[B27] Kesavardhana S. Malireddi R. K. S. Kanneganti T. D. (2020b). Caspases in cell death, inflammation, and pyroptosis. Annu. Rev. Immunol. 38, 567–595. doi: 10.1146/annurev-immunol-073119-095439, PMID: 32017655 PMC7190443

[B28] Kobayashi K. S. van den Elsen P. J. (2012). NLRC5: a key regulator of MHC class I-dependent immune responses. Nat. Rev. Immunol. 12, 813–820. doi: 10.1038/nri3339, PMID: 23175229

[B29] Kumar R. Singh P. Kolloli A. Shi L. Bushkin Y. Tyagi S. . (2019). Immunometabolism of phagocytes during mycobacterium tuberculosis infection. Front. Mol. Biosci. 6. doi: 10.3389/fmolb.2019.00105, PMID: 31681793 PMC6803600

[B30] Kuriakose T. Man S. M. Malireddi R. K. Karki R. Kesavardhana S. Place D. E. . (2016). ZBP1/DAI is an innate sensor of influenza virus triggering the NLRP3 inflammasome and programmed cell death pathways. Sci. Immunol. 1, aag2045. doi: 10.1126/sciimmunol.aag2045, PMID: 27917412 PMC5131924

[B31] Lam A. Prabhu R. Gross C. M. Riesenberg L. A. Singh V. Aggarwal S. (2017). Role of apoptosis and autophagy in tuberculosis. Am. J. Physiol. Lung Cell Mol. Physiol. 313, L218–l229. doi: 10.1152/ajplung.00162.2017, PMID: 28495854 PMC5582934

[B32] Langlais D. Barreiro L. B. Gros P. (2016). The macrophage IRF8/IRF1 regulome is required for protection against infections and is associated with chronic inflammation. J. Exp. Med. 213, 585–603. doi: 10.1084/jem.20151764, PMID: 27001747 PMC4821649

[B33] Lee J. Boyce S. Powers J. Baer C. Sassetti C. M. Behar S. M. (2020). CD11cHi monocyte-derived macrophages are a major cellular compartment infected by Mycobacterium tuberculosis. PLoS Pathog. 16, e1008621. doi: 10.1371/journal.ppat.1008621, PMID: 32544188 PMC7319360

[B35] Lee S. Karki R. Wang Y. Nguyen L. N. Kalathur R. C. Kanneganti T. D. (2021). AIM2 forms a complex with pyrin and ZBP1 to drive PANoptosis and host defence. Nature 597, 415–419. doi: 10.1038/s41586-021-03875-8, PMID: 34471287 PMC8603942

[B34] Lee J. Remold H. G. Ieong M. H. Kornfeld H. (2006). Macrophage apoptosis in response to high intracellular burden of Mycobacterium tuberculosis is mediated by a novel caspase-independent pathway. J. Immunol. 176, 4267–4274. doi: 10.4049/jimmunol.176.7.4267, PMID: 16547264

[B36] Levine A. J. (1997). p53, the cellular gatekeeper for growth and division. Cell 88, 323–331. doi: 10.1016/s0092-8674(00)81871-1, PMID: 9039259

[B37] Lin J. Chang Q. Dai X. Liu D. Jiang Y. Dai Y. (2019). Early secreted antigenic target of 6-kDa of Mycobacterium tuberculosis promotes caspase-9/caspase-3-mediated apoptosis in macrophages. Mol. Cell Biochem. 457, 179–189. doi: 10.1007/s11010-019-03522-x, PMID: 30911956

[B38] Liu Y. Fan C. Zhang Y. Yu X. Wu X. Zhang X. . (2017). RIP1 kinase activity-dependent roles in embryonic development of Fadd-deficient mice. Cell Death Differ 24, 1459–1469. doi: 10.1038/cdd.2017.78, PMID: 28574501 PMC5520462

[B39] Lv J. He X. Wang H. Wang Z. Kelly G. T. Wang X. . (2017). TLR4-NOX2 axis regulates the phagocytosis and killing of Mycobacterium tuberculosis by macrophages. BMC Pulm Med. 17, 194. doi: 10.1186/s12890-017-0517-0, PMID: 29233104 PMC5727946

[B40] Malik A. Kanneganti T. D. (2017). Inflammasome activation and assembly at a glance. J. Cell Sci. 130, 3955–3963. doi: 10.1242/jcs.207365, PMID: 29196474 PMC5769591

[B41] Malireddi R. K. S. Bynigeri R. R. Mall R. Nadendla E. K. Connelly J. P. Pruett-Miller S. M. . (2023). Whole-genome CRISPR screen identifies RAVER1 as a key regulator of RIPK1-mediated inflammatory cell death, PANoptosis. iScience 26, 106938. doi: 10.1016/j.isci.2023.106938, PMID: 37324531 PMC10265528

[B42] Malireddi R. K. S. Gurung P. Kesavardhana S. Samir P. Burton A. Mummareddy H. . (2020a). Innate immune priming in the absence of TAK1 drives RIPK1 kinase activity-independent pyroptosis, apoptosis, necroptosis, and inflammatory disease. J. Exp. Med. 217, jem.20191644. doi: 10.1084/jem.20191644, PMID: 31869420 PMC7062518

[B43] Malireddi R. K. S. Kesavardhana S. Kanneganti T. D. (2019). ZBP1 and TAK1: master regulators of NLRP3 inflammasome/pyroptosis, apoptosis, and necroptosis (PAN-optosis). Front. Cell Infect. Microbiol. 9, jem.20191644. doi: 10.3389/fcimb.2019.00406, PMID: 31850239 PMC6902032

[B44] Malireddi R. K. S. Kesavardhana S. Karki R. Kancharana B. Burton A. R. Kanneganti T. D. (2020b). RIPK1 distinctly regulates yersinia-induced inflammatory cell death, PANoptosis. Immunohorizons 4, 789–796. doi: 10.4049/immunohorizons.2000097, PMID: 33310881 PMC7906112

[B45] Malireddi R. K. S. Tweedell R. E. Kanneganti T. D. (2020c). PANoptosis components, regulation, and implications. Aging (Albany NY) 12, 11163–11164. doi: 10.18632/aging.103528, PMID: 32575071 PMC7343493

[B46] Marakalala M. J. Martinez F. O. Plüddemann A. Gordon S. (2018). Macrophage heterogeneity in the immunopathogenesis of tuberculosis. Front. Microbiol. 9. doi: 10.3389/fmicb.2018.01028, PMID: 29875747 PMC5974223

[B47] Mohanty S. Dal Molin M. Ganguli G. Padhi A. Jena P. Selchow P. . (2016). Mycobacterium tuberculosis EsxO (Rv2346c) promotes bacillary survival by inducing oxidative stress mediated genomic instability in macrophages. Tuberculosis (Edinb) 96, 44–57. doi: 10.1016/j.tube.2015.11.006, PMID: 26786654

[B48] Mukherjee S. Keitany G. Li Y. Wang Y. Ball H. L. Goldsmith E. J. . (2006). Yersinia YopJ acetylates and inhibits kinase activation by blocking phosphorylation. Science 312, 1211–1214. doi: 10.1126/science.1126867, PMID: 16728640

[B49] Nagata M. Carvalho Schäfer Y. Wachsmuth L. Pasparakis M. (2024). A shorter splicing isoform antagonizes ZBP1 to modulate cell death and inflammatory responses. EMBO J. 43, 5037–5056. doi: 10.1038/s44318-024-00238-7, PMID: 39300211 PMC11535224

[B50] Nagata S. Tanaka M. (2017). Programmed cell death and the immune system. Nat. Rev. Immunol. 17, 333–340. doi: 10.1038/nri.2016.153, PMID: 28163302

[B51] Nassour J. Aguiar L. G. Correia A. Schmidt T. T. Mainz L. Przetocka S. . (2023). Telomere-to-mitochondria signalling by ZBP1 mediates replicative crisis. Nature 614, 767–773. doi: 10.1038/s41586-023-05710-8, PMID: 36755096 PMC9946831

[B52] Nguyen L. N. Kanneganti T. D. (2022). PANoptosis in viral infection: the missing puzzle piece in the cell death field. J. Mol. Biol. 434, 167249. doi: 10.1016/j.jmb.2021.167249, PMID: 34537233 PMC8444475

[B53] Nie X. Miao S. Hou Y. Ma Y. Li M. Liu Y. . (2025). TLR4-mediated endoplasmic reticulum stress regulates pyroptosis in macrophages infected with the Bacillus Calmette-Guérin mycobacterial. Int. Immunopharmacol 152, 114346. doi: 10.1016/j.intimp.2025.114346, PMID: 40064059

[B54] Park J. S. Tamayo M. H. Gonzalez-Juarrero M. Orme I. M. Ordway D. J. (2006). Virulent clinical isolates of Mycobacterium tuberculosis grow rapidly and induce cellular necrosis but minimal apoptosis in murine macrophages. J. Leukoc. Biol. 79, 80–86. doi: 10.1189/jlb.0505250, PMID: 16275894

[B55] Patterson J. B. Samuel C. E. (1995). Expression and regulation by interferon of a double-stranded-RNA-specific adenosine deaminase from human cells: evidence for two forms of the deaminase. Mol. Cell Biol. 15, 5376–5388. doi: 10.1128/mcb.15.10.5376, PMID: 7565688 PMC230787

[B56] Ramakrishnan L. (2012). Revisiting the role of the granuloma in tuberculosis. Nat. Rev. Immunol. 12, 352–366. doi: 10.1038/nri3211, PMID: 22517424

[B57] Roca F. J. Ramakrishnan L. (2013). TNF dually mediates resistance and susceptibility to mycobacteria via mitochondrial reactive oxygen species. Cell 153, 521–534. doi: 10.1016/j.cell.2013.03.022, PMID: 23582643 PMC3790588

[B58] Sharma B. R. Karki R. Rajesh Y. Kanneganti T. D. (2023). Immune regulator IRF1 contributes to ZBP1-, AIM2-, RIPK1-, and NLRP12-PANoptosome activation and inflammatory cell death (PANoptosis). J. Biol. Chem. 299, 105141. doi: 10.1016/j.jbc.2023.105141, PMID: 37557956 PMC10494469

[B59] Shen J. Fu Y. Liu F. Wu J. Zhang H. Sun J. . (2025). Salvianolic acid B inhibits ZBP1-mediated PANoptosis in mycobacterium tuberculosis-infected macrophages by targeting TNFR1. Phytother. Res. 39, 4028–4045. doi: 10.1002/ptr.70042, PMID: 40717039

[B60] Shen P. Li Q. Ma J. Tian M. Hong F. Zhai X. . (2017). IRAK-M alters the polarity of macrophages to facilitate the survival of Mycobacterium tuberculosis. BMC Microbiol. 17, 185. doi: 10.1186/s12866-017-1095-2, PMID: 28835201 PMC5569470

[B61] Shi G. Mao G. Xie K. Wu D. Wang W. (2018). MiR-1178 regulates mycobacterial survival and inflammatory responses in Mycobacterium tuberculosis-infected macrophages partly via TLR4. J. Cell Biochem. 119, 7449–7457. doi: 10.1002/jcb.27054, PMID: 29781535

[B62] Shi J. Zhao Y. Wang K. Shi X. Wang Y. Huang H. . (2015). Cleavage of GSDMD by inflammatory caspases determines pyroptotic cell death. Nature 526, 660–665. doi: 10.1038/nature15514, PMID: 26375003

[B63] Stanger B. Z. Leder P. Lee T. H. Kim E. Seed B. (1995). RIP: a novel protein containing a death domain that interacts with Fas/APO-1 (CD95) in yeast and causes cell death. Cell 81, 513–523. doi: 10.1016/0092-8674(95)90072-1, PMID: 7538908

[B64] Stutz M. D. Ojaimi S. Allison C. Preston S. Arandjelovic P. Hildebrand J. M. . (2018). Necroptotic signaling is primed in Mycobacterium tuberculosis-infected macrophages, but its pathophysiological consequence in disease is restricted. Cell Death Differ 25, 951–965. doi: 10.1038/s41418-017-0031-1, PMID: 29229989 PMC5943269

[B65] Sun X. Yin J. Starovasnik M. A. Fairbrother W. J. Dixit V. M. (2002). Identification of a novel homotypic interaction motif required for the phosphorylation of receptor-interacting protein (RIP) by RIP3. J. Biol. Chem. 277, 9505–9511. doi: 10.1074/jbc.M109488200, PMID: 11734559

[B66] Sundaram B. Kanneganti T. D. (2021). Advances in understanding activation and function of the NLRC4 inflammasome. Int. J. Mol. Sci. 22, 1048. doi: 10.3390/ijms22031048, PMID: 33494299 PMC7864484

[B67] Sundaram B. Karki R. Kanneganti T. D. (2022). NLRC4 deficiency leads to enhanced phosphorylation of MLKL and necroptosis. Immunohorizons 6, 243–252. doi: 10.4049/immunohorizons.2100118, PMID: 35301258 PMC8996759

[B68] Sundaram B. Pandian N. Kim H. J. Abdelaal H. M. Mall R. Indari O. . (2024). NLRC5 senses NAD(+) depletion, forming a PANoptosome and driving PANoptosis and inflammation. Cell 187, 4061–4077.e4017. doi: 10.1016/j.cell.2024.05.034, PMID: 38878777 PMC11283362

[B69] Sundaram B. Pandian N. Mall R. Wang Y. Sarkar R. Kim H. J. . (2023). NLRP12-PANoptosome activates PANoptosis and pathology in response to heme and PAMPs. Cell 186, 2783–2801.e2720. doi: 10.1016/j.cell.2023.05.005, PMID: 37267949 PMC10330523

[B70] Theobald S. J. Müller T. A. Lange D. Keck K. Rybniker J. (2024). The role of inflammasomes as central inflammatory hubs in Mycobacterium tuberculosis infection. Front. Immunol. 15. doi: 10.3389/fimmu.2024.1436676, PMID: 39324136 PMC11422116

[B71] Tobin D. M. Roca F. J. Oh S. F. McFarland R. Vickery T. W. Ray J. P. . (2012). Host genotype-specific therapies can optimize the inflammatory response to mycobacterial infections. Cell 148, 434–446. doi: 10.1016/j.cell.2011.12.023, PMID: 22304914 PMC3433720

[B72] Tsuchiya K. Nakajima S. Hosojima S. Thi Nguyen D. Hattori T. Manh Le T. . (2019). Caspase-1 initiates apoptosis in the absence of gasdermin D. Nat. Commun. 10, 2091. doi: 10.1038/s41467-019-09753-2, PMID: 31064994 PMC6505044

[B73] Tweedell R. E. Kanneganti T. D. (2020). Advances in inflammasome research: recent breakthroughs and future hurdles. Trends Mol. Med. 26, 969–971. doi: 10.1016/j.molmed.2020.07.010, PMID: 32948447 PMC7678016

[B74] Van Opdenbosch N. Van Gorp H. Verdonckt M. Saavedra P. H. V. de Vasconcelos N. M. Gonçalves A. . (2017). Caspase-1 engagement and TLR-induced c-FLIP expression suppress ASC/caspase-8-dependent apoptosis by inflammasome sensors NLRP1b and NLRC4. Cell Rep. 21, 3427–3444. doi: 10.1016/j.celrep.2017.11.088, PMID: 29262324 PMC5746600

[B75] Varadi M. Bertoni D. Magana P. Paramval U. Pidruchna I. Radhakrishnan M. . (2024). AlphaFold Protein Structure Database in 2024: providing structure coverage for over 214 million protein sequences. Nucleic Acids Res. 52, D368–d375. doi: 10.1093/nar/gkad1011, PMID: 37933859 PMC10767828

[B76] Varghese G. P. Uporova L. Halfvarson J. Sirsjö A. Fransén K. (2015). Polymorphism in the NLRP3 inflammasome-associated EIF2AK2 gene and inflammatory bowel disease. Mol. Med. Rep. 11, 4579–4584. doi: 10.3892/mmr.2015.3236, PMID: 25607115

[B77] Velmurugan K. Chen B. Miller J. L. Azogue S. Gurses S. Hsu T. . (2007). Mycobacterium tuberculosis nuoG is a virulence gene that inhibits apoptosis of infected host cells. PloS Pathog. 3, e110. doi: 10.1371/journal.ppat.0030110, PMID: 17658950 PMC1924871

[B78] Walsh J. G. Logue S. E. Lüthi A. U. Martin S. J. (2011). Caspase-1 promiscuity is counterbalanced by rapid inactivation of processed enzyme. J. Biol. Chem. 286, 32513–32524. doi: 10.1074/jbc.M111.225862, PMID: 21757759 PMC3173193

[B79] Wang Y. Gao W. Shi X. Ding J. Liu W. He H. . (2017). Chemotherapy drugs induce pyroptosis through caspase-3 cleavage of a gasdermin. Nature 547, 99–103. doi: 10.1038/nature22393, PMID: 28459430

[B80] Wang Y. Kanneganti T. D. (2021). From pyroptosis, apoptosis and necroptosis to PANoptosis: A mechanistic compendium of programmed cell death pathways. Comput. Struct. Biotechnol. J. 19, 4641–4657. doi: 10.1016/j.csbj.2021.07.038, PMID: 34504660 PMC8405902

[B81] Wei S. Wu L. Xiang Z. Yang X. Pei D. Jiang L. . (2024). EIF2AK2 protein targeted activation of AIM2-mediated PANoptosis promotes sepsis-induced acute kidney injury. Ren Fail 46, 2403649. doi: 10.1080/0886022x.2024.2403649, PMID: 39311631 PMC11421145

[B82] World Health, O (2024). Global tuberculosis report 2024 (Geneva: World Health Organization).

[B83] Wu Y. H. Kuo W. C. Wu Y. J. Yang K. T. Chen S. T. Jiang S. T. . (2014). Participation of c-FLIP in NLRP3 and AIM2 inflammasome activation. Cell Death Differ 21, 451–461. doi: 10.1038/cdd.2013.165, PMID: 24270411 PMC3921593

[B84] Xie M. Yu Y. Kang R. Zhu S. Yang L. Zeng L. . (2025). Author Correction: PKM2-dependent glycolysis promotes NLRP3 and AIM2 inflammasome activation. Nat. Commun. 16, 6158. doi: 10.1038/s41467-025-60997-7, PMID: 40615383 PMC12227756

[B85] Xu W. Che Y. Zhang Q. Huang H. Ding C. Wang Y. . (2021). Apaf-1 pyroptosome senses mitochondrial permeability transition. Cell Metab. 33, 424–436.e410. doi: 10.1016/j.cmet.2020.11.018, PMID: 33308446

[B86] Yang S. Li F. Jia S. Zhang K. Jiang W. Shang Y. . (2015). Early secreted antigen ESAT-6 of Mycobacterium Tuberculosis promotes apoptosis of macrophages via targeting the microRNA155-SOCS1 interaction. Cell Physiol. Biochem. 35, 1276–1288. doi: 10.1159/000373950, PMID: 25721573

[B87] Yang Z. H. Wu X. N. He P. Wang X. Wu J. Ai T. . (2020). A non-canonical PDK1-RSK signal diminishes pro-caspase-8-mediated necroptosis blockade. Mol. Cell 80, 296–310.e296. doi: 10.1016/j.molcel.2020.09.004, PMID: 32979304

[B88] Yuan J. Amin P. Ofengeim D. (2019). Necroptosis and RIPK1-mediated neuroinflammation in CNS diseases. Nat. Rev. Neurosci. 20, 19–33. doi: 10.1038/s41583-018-0093-1, PMID: 30467385 PMC6342007

[B89] Zhai W. Wu F. Zhang Y. Fu Y. Liu Z. (2019). The immune escape mechanisms of mycobacterium tuberculosis. Int. J. Mol. Sci. 20, 340. doi: 10.3390/ijms20020340, PMID: 30650615 PMC6359177

[B90] Zhang W. Lu Q. Dong Y. Yue Y. Xiong S. (2018). Rv3033, as an emerging anti-apoptosis factor, facilitates mycobacteria survival via inhibiting macrophage intrinsic apoptosis. Front. Immunol. 9. doi: 10.3389/fimmu.2018.02136, PMID: 30319611 PMC6168788

[B91] Zhang Y. Xu D. Nie Q. Wang J. Fang D. Xie Y. . (2024). Macrophages exploit the mannose receptor and JAK-STAT1-MHC-II pathway to drive antigen presentation and the antimycobacterial immune response after BCG vaccination. Acta Biochim. Biophys. Sin. (Shanghai) 56, 1130–1144. doi: 10.3724/abbs.2024100, PMID: 38894685 PMC11399420

[B92] Zheng M. Kanneganti T. D. (2020a). Newly identified function of caspase-6 in ZBP1-mediated innate immune responses, NLRP3 inflammasome activation, PANoptosis, and host defense. J. Cell Immunol. 2, 341–347. doi: 10.33696/immunology.2.064, PMID: 33426542 PMC7793005

[B93] Zheng M. Kanneganti T. D. (2020b). The regulation of the ZBP1-NLRP3 inflammasome and its implications in pyroptosis, apoptosis, and necroptosis (PANoptosis). Immunol. Rev. 297, 26–38. doi: 10.1111/imr.12909, PMID: 32729116 PMC7811275

[B94] Zheng M. Karki R. Vogel P. Kanneganti T. D. (2020). Caspase-6 is a key regulator of innate immunity, inflammasome activation, and host defense. Cell 181, 674–687.e613. doi: 10.1016/j.cell.2020.03.040, PMID: 32298652 PMC7425208

[B95] Zhou X. Yang J. Zhang Z. Zhang L. Lie L. Zhu B. . (2019). Interferon regulatory factor 1 eliminates mycobacteria by suppressing p70 S6 kinase via mechanistic target of rapamycin signaling. J. Infect. 79, 262–276. doi: 10.1016/j.jinf.2019.06.007, PMID: 31226272

